# Modified-Distribution Entropy as the Features for the Detection of Epileptic Seizures

**DOI:** 10.3389/fphys.2020.00607

**Published:** 2020-06-25

**Authors:** Si Thu Aung, Yodchanan Wongsawat

**Affiliations:** Department of Biomedical Engineering, Faculty of Engineering, Mahidol University, Salaya, Thailand

**Keywords:** distribution entropy, electroencephalogram (EEG), entropy, epilepsy, fuzzy entropy

## Abstract

Epilepsy is one of the most common chronic neurological disorders, and therefore, diagnosis and treatment methods are urgently needed for these patients. Many methods and algorithms that can detect seizures in epileptic patients have been proposed. Electroencephalogram (EEG) is one of helpful tools for investigating epilepsy forms in patients, however, an expert in the neurological field must perform a visual inspection to identify a seizure. Such analyses require longer time because of the huge dataset recorded from many electrodes which are put on the human scalp. With the non-stationary nature of EEG, especially during the abnormality periods, entropy measures gain more interest in the field. In this work, by exploring the advantages of both reliable state-of-the-art entropies, fuzzy entropy and distribution entropy, a modified-Distribution entropy (mDistEn) for epilepsy detection is proposed. As the results, the proposed mDistEn method can successfully achieve the same consistency and better accuracy than using the state-of-the-art entropies. The mDistEn corresponds to higher Area Under the Curve (AUC) values compared with the fuzzy entropy and the distribution entropy and yields 92% classification accuracy.

## Introduction

According to the World Health Organization (WHO), ~50 million of people suffer from epilepsy and about 10% of the population of the world has once had a seizure in their daily routine (Epilepsy-information, 2019). Moreover, there are nearly 15 million people with epilepsy in Asian countries. Roughly 1% of the people who live in these regions and including patients with epilepsy visit faith healers rather than medical doctors, and only 10–20% of all patients with epilepsy receive appropriate treatment. Nonetheless, 70–80% of people with epilepsy can lead normal lives if properly treated; therefore, it should be critically considered why 80–90% of people with epilepsy are not appropriately treated (Media-center, 2011). The brain acts as a control center that commands all movements and responses including voluntary and involuntary responses of the body. Electrical activity in the brain is used for communication via nerve cells but abnormal signals received by the brain may interrupt normal function and result in a seizure (Health, 2019). Epilepsy is a chronic neurological disorder that may cause movement disturbance, loss of awareness or sensation, and disrupted mood or mental function; therefore, diagnosis and treatment are of major importance for epilepsy patients (Kaya et al., 2014).

Electroencephalogram (EEG) which is a potential method that can be used not only for detection but also for prediction of epileptic seizures according to extensive evidence (Myers and Kozma, 2018; Li et al., 2019). EEG does not require open surgery and, thus, is a safe, non-invasive testing procedure that can yield a huge amount of information regarding the health of the patient (Ocak, 2009). In an epileptic patient, EEG tests can be performed by using electrodes placed on the affected area of the human scalp to record brain signals for analysis (Coyle et al., 2010). However, the recorded EEG signals must be visually inspected by an expert in this field, and such tests take longer time than an automatic method because of the extensive amount of data (Gandhi et al., 2010).

According to the literature reviews, there are various contributions on designing the efficient feature extraction methods for epileptic seizure detection, e.g., empirical mode decomposition (EMD) (Pachori, 2008; Bajaj and Pachori, 2011; Pachori and Bajaj, 2011; Pachori and Patidar, 2014; Pachori et al., 2015; Agrawal et al., 2019), time-frequency representation (Bhati et al., 2017, 2020a,b; Sharma and Pachori, 2017; de la Serna et al., 2020; Gupta et al., 2020; Nishad and Pachori, 2020), phase representation (Sharma and Pachori, 2015), deep neural network (Sharma et al., 2020a,b), fractional order modeling (Joshi et al., 2014), and local binary pattern (Kumar et al., 2015; Tiwari et al., 2016).

Complexity can be measured by different methods and can compare time series to distinguish regular, chaotic and random behavior (Paluš, 1998). Claude Shannon developed the modern concept of “information” or “logical” entropy as part of information theory in the late 1940s (Shannon, 1948). With the non-stationary nature of EEG, especially during the abnormality periods, entropy measures gain more interest in the field. There are many of entropy methods, such as Bhattacharyya et al. (2017), Sharma et al. (2018, 2019), Gupta and Pachori (2019), approximate entropy (ApEN) (Pincus et al., 1991), sample entropy (SampEN) (Richman and Moorman, 2000), permutation entropy (PermEN) (Bandt and Pompe, 2002), distribution entropy (DistEn) (Li et al., 2015a), fuzzy distribution entropy (fDistEn) (Zhang et al., 2018), and these methods have been proposed to examine physiological time series data in recent years. Among these entropies, fuzzy entropy and distribution entropy reveal the promising results (Li et al., 2018). However, both of them give the promising results in different types of epileptic seizures data. By exploring the advantages of both fuzzy entropy and distribution entropy, a modified-Distribution entropy (mDistEn) is proposed for the detection of epileptic seizures.

This paper is divided into three sections. The following section presents the data analyzed and describes the detail of the methods applied in this paper. The second section presents and explains about the results and discuss their meanings. The last section of the paper is the conclusion and future direction of our entropy method.

## Materials and Methods

### EEG Data for Analysis

**Figure 1:**
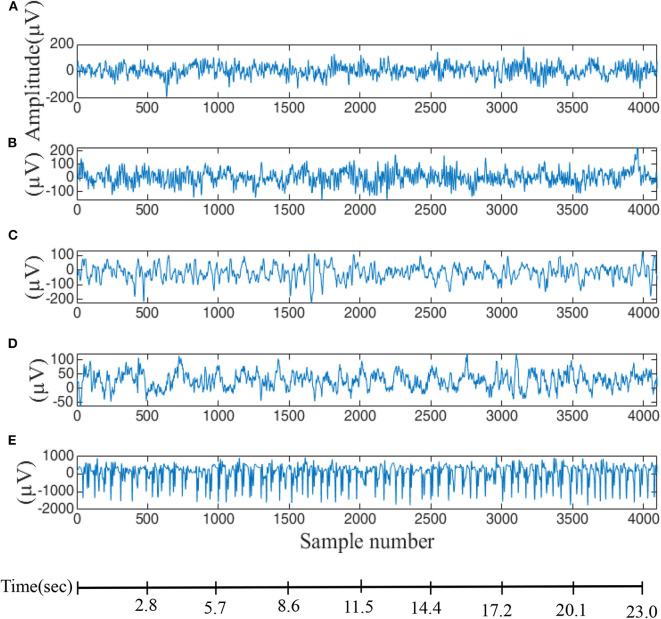
EEG data used for evaluation of difference entropy algorithms. **(A,B)** Are data from five healthy volunteers with eyes-opened and eyes-closed. **(C)** Data recorded from patients before an epileptic attack, **(D)** from the epileptic zone, and **(E)** during an epileptic attack.

EEG seizure data is available from the University of Bonn (Andrzejak et al., 2001a,b) which provides a free and reliable database for analysis of all types of methods that are related to seizure activity. Five sets of data are included (A, B, C, D, E) corresponding to eye-closed and eye-opened states of healthy subjects (two classes—A and B) and, the interictal period (two classes—C and D) and ictal period (one class—E) of epilepsy patients. These five data-sets contain a single-channel electrode with 100 EEG segments and each segment is 23.6 s long (4,096 sampling points with a sampling rate of 173.61 Hz), as displayed in Figure 1. In this paper, fuzzy entropy and distribution entropy are calculated for comparison with the modified entropy calculation, i.e., the calculation based on distribution entropy and combined with some parameters from fuzzy entropy. First, EEG signals are used to reconstruct the state-space using the embedding dimension and then the vector from the state-space is ranked according to a fuzzy membership function. The last step is the calculation of the FuzzyEn value, as described in the next subsection.

**Figure 2:**
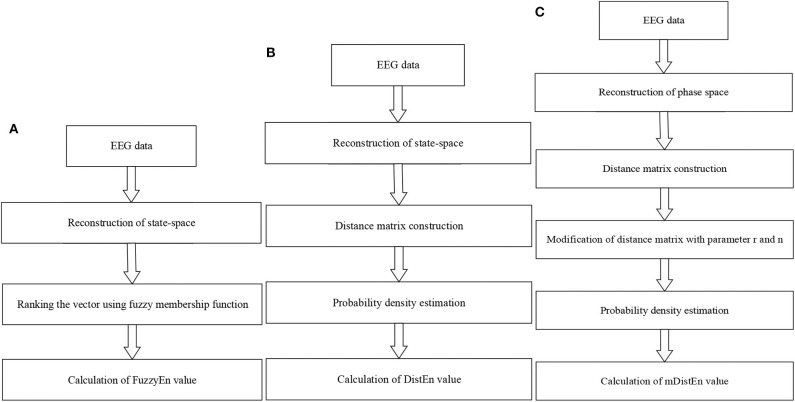
Flow diagrams for calculating the entropy values. **(A)** Fuzzy entropy calculation; **(B)** distribution entropy calculation; and **(C)** modified-distribution entropy calculation.

The mDistEn is calculated from a reconstruction of the state-space similar to fuzzy entropy. However, the difference between FuzzyEn and mDistEn is the construction of a distance matrix in the second step. The modified-distribution entropy is also evaluated by reconstruction of the phase space, i.e., it is also the state-space which is the representation of the behavior of a system in the geometric form (Yadid and Friedman, 2008). Next, the empirical Probability Density Function (ePDF) is estimated to obtain the probability of the distance matrix. The following steps are the same with the DistEn except that the parameter values *r* (0.2 × standard deviation of all dataset) and *n* = 2 are included before calculating the ePDF. These modifications provide the better discrimination of the ictal state from the normal and interictal states in epilepsy patient. Flow charts of these three algorithms are shown in Figures 2A–C.

### Fuzzy Entropy (FuzzyEn)

Approximation entropy and sample entropy that can measure the similarity of a vector using a Heaviside function, given by:

(1) $$\begin{array}{l} \theta \left(z\right)=\;\{\begin{array}{cc} 1,\; & if\;z\geq 0\\ 0,\; & if\;z<0 \end{array} \end{array}$$

This kind of function is a conventional two-state classifier, which enables justifying the belongingness using a given class (Chen et al., 2007). Given a time series with *N* data points, {*x* (n)} = *x* (1), *x* (2), …, *x* (*N*), the following algorithm can be used to calculate FuzzyEn (Xiang et al., 2015):

- For 1 ≤ *i* ≤ *N* – *m*+1, where *m* is given, form a vector sequence {${\text{X}}_{i}^{m}\;$(1) … ${\text{X}}_{i}^{m}\;$(*N* – *m* + 1)}, which is defined as
  (2) $$\begin{array}{l} {\text{X}}_{i}^{m}\;=\;\left\{x\;\left(i\right),\;x\;\left(i+1\right),\ldots ,\;x\;\left(i\;+\;m\;1\right)\right\}\;-\;x_{0}\;(i), \end{array}$$
  where ${\text{X}}_{i}^{m}$ is *m* consecutive *x* values, commencing with the *i*th point and that needs to be generalized by eliminating a baseline
  (3) $$\begin{array}{l} x_{0}\left(i\right)=\;\frac{1}{m}\sum_{j=0}^{m-1}x\left(i+j\right). \end{array}$$
- Define the distance between **X**^*m*^ (*i*) and **X**^*m*^ (*j*) (1 ≤ *i, j* ≤ (*N* – *m*), *i* ≠ *j*) as the maximum absolute difference of the corresponding components
  (4) $$\begin{array}{l} d_{i}j^{m}=d[X_{i}^{m},X_{j}^{m}]=max_{\left(k\in (0,m-1)\right)}|x(i+k)-x_{0}(i)\\ \;-(x(j+k)-x_{0}(j))|. \end{array}$$
- Calculate the similarity degree $D_{ij}^{m}$ by using *n* and the *r* value through a fuzzy function
  (5) $$\begin{array}{l} D_{ij}^{m}\;\left(n,r\right)=\text{ μ}\left(d_{ij}^{m},n,r\right)=\exp\left(\frac{-{\left(d_{ij}^{m}\right)}^{2}}{r}\right).\; \end{array}$$
- Define the function ∅^*m*^ as
  (6) $$\begin{array}{l} \emptyset^{m}\left(n,r\right)=\;\frac{1}{N-m}\;\sum_{i=1}^{N-m}\left(\frac{1}{N-m-1}\sum_{j=1,j\neq i}^{N-m}D_{ij}^{m}\right). \end{array}$$
- Similarly, **X**^m+1^ (*i*) vector form, the value *m* can be increased to *m*+1 and then, the function ∅^*m*+1^ as
  (7) $$\begin{array}{l} \emptyset^{m+1}\left(n,r\right)=\;\frac{1}{N-m}\;\sum_{i=1}^{N-m}\left(\frac{1}{N-m-1}\sum_{j=1,j\neq i}^{N-m}D_{ij}^{m+1}\right). \end{array}$$
- Finally, a time series with finite data, can be evaluated by the following equation
  (8) $$\begin{array}{l} \text{FuzzyEn }\left(m,n,r,N\right)=\ln\emptyset^{m\;}\left(n,r\right)-\ln\emptyset^{m+1\;}(n,r). \end{array}$$

### Distribution Entropy (DistEn)

Distribution entropy is an entropy that measures the complexity of time series data using the empirical probability density function (ePDF) of distances for inter-vectors in the state space (Li et al., 2015a). Given a time-series {*x* (*i*), 1 ≤ *i* ≤ *N*} for all *N* points, the distribution entropy (DistEn) can be estimated by the steps below (Li et al., 2016):

- State-space reconstruction can be completed by forming *N – (m –* 1) × τ vectors **X** (*i*) using **X** (*i*) = {*x* (*i*), *x (i*+1), …, *x (i* +(*m –* 1) × τ)}, where 1 ≤ i ≤ *N – (m –* 1) × τ and, *m* is the embedding dimension and τ is time delay.
- Distance matrix construction used to compute the distances between all possible combinations of **X** (*i*) and **X** (*j*) by
  (9) $$\begin{array}{l} d_{ij}=\;\max\;\{|x\;(i\;+\;k)\text{ x }(j\;+\;k)|,\;0\leq k\leq m\;1\}\text{ for all}\\ 1\leq i,\;j\;\leq \;m\;1. \end{array}$$
  The distance matrix **D** = {*d*_*ij*_} is defined. Then, the ePDF is calculated using a histogram with bin numbers, {P_*t*_, *t* = 1, 2, 3, …, *B*}.
- The final step is the calculation of the distribution entropy.
  (10) $$\begin{array}{l} \text{Dist}En\;\left(m,B\right)=\;-\frac{1}{\log_{2}\left(B\right)}\sum_{t=1}^{B}P_{t}\log_{2}\left(P_{t}\right). \end{array}$$

### Modified-Distribution Entropy (mDistEn)

A new method, which is implemented based on distribution entropy, is the addition of two more threshold parameters “*r*” and “*n*” to existing parameters. Among these two parameters, *r* is set by multiplying to the standard deviation of all data values by 0.2 and *n* is set to 2. For a given time series *N* sample,

- For phase-space reconstruction, create *N-(m*-1) × τ vector **X** (*i*) by *x* (*i*) = {*x* (*i*) + *x* (*i*+1), …, *x* (*i* + (*m* – 1) × τ)}, where *m* is the embedding dimension and τ is the time delay.
- Computes a distance matrix (**D**_*ij*_) between **X** (*i*) and **X** (*j*) (1 ≤ *i, j* ≤ *m* – *1, i* ≠ *j*) using the Euclidean method.
- In this step, **D**_mat_ is divided by *r* and squared (*n* = 2):
  (11) $$\begin{array}{l} {\text{D}}_{mat}=\;{\left(\frac{{\text{D}}_{ij}}{r}\right)}^{n}. \end{array}$$
- After obtaining **D**_mat_, the ePDF is calculated using the histogram approach from the **D**_mat_ from the previous steps with the bin number. The probability for that number can be given as {*P*_*t*_, *t* = 1, 2, 3, …, *B*}.
- mDistEn can be described as follows:
  (12) $$\begin{array}{l} mDistEn\;\left(m,\tau ,r,n,B\right)=\;-\frac{1}{\log_{2}\left(B\right)}\sum_{t=1}^{B}P_{t}\left({\text{D}}_{mat}\right)\\ \log_{2}\left[P_{t}\left({\text{D}}_{mat}\right)\right]. \end{array}$$

#### Data Visualization of mDistEn

**Figure 3:**
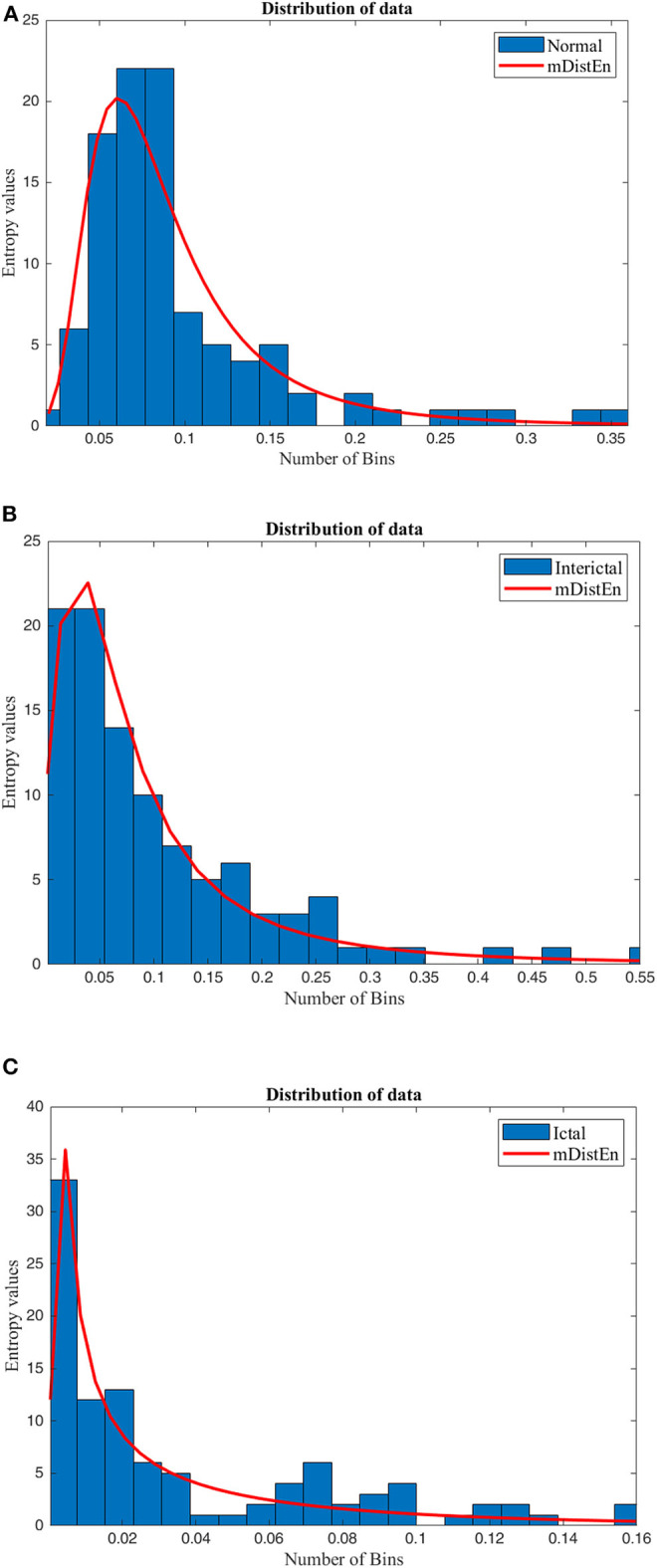
Distribution of the data. The mDistEn is calculated using **(A)** normal EEG datasets A and B and its distribution, **(B)** the interictal datasets C and D and its distribution, and **(C)** ictal dataset E and its distribution.

The data obtained from the result of mDistEn is visualized according to the shape of the distribution and is shown in Figure 3 and (A) mDistEn is calculated using normal EEG data and the distribution. Figure 3B shows the result of the interictal data, which is a combination of EEG datasets C and D and Figure 3C shows the onset seizure data.

#### Parameter Selection

The values of the gradient of the boundary (*n*) and the width (*r*) of the exponential function applied in the fuzzy entropy, are *n* = 2 and *r* = 0.2 × standard deviation of the time series (Chen et al., 2007). These values are not only used in fuzzy entropy but also used in the calculation of mDistEn. Moreover, the embedding dimension (*m*) and the time delay (τ) used in the calculation are the same values of *m* = 3 and τ = 1, respectively (Li et al., 2015a). Finally, the bin value (*B* = 64) is used for estimation of the two distribution entropies; DistEn (Li et al., 2016) and mDistEn.

## Results and Discussion

### Analysis With Theoretical Data

**Figure 4:**
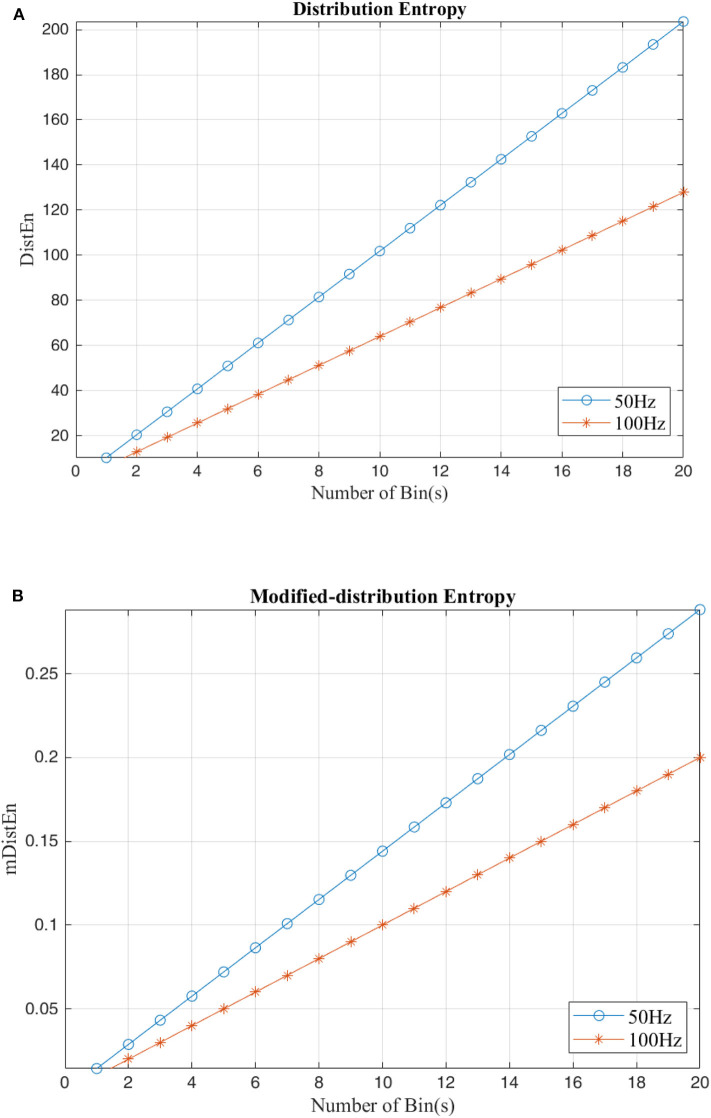
Calculation of distribution entropy and the modified distribution entropy using a sinusoidal signal with difference frequencies (50 and 100 Hz). **(A)** Distribution entropy. **(B)** Modified-Distribution entropy.

Both the distribution entropy and the modified-distribution entropy are simulated using periodic sinusoidal signals with frequencies of 50 and 100 Hz. The length of the signal is 2 s long, and the sampling rate is 0.5 kHz. Since calculation of the distribution entropies depends on the parameter values, the number of values in each bin must range from 50 to 1,000 per bin, and the number of bins is increased to 50 bins for testing the stability. According to the figures, both the distribution entropy and the modified-distribution entropy have the same consistency in measurements (Li et al., 2015b). Some parameters are added to mDistEn but it still has strong regularity even when testing different frequencies. Figures 4A,B shows the result of the simulation using waves based on the estimation of different distribution entropies.

### Analysis With Experimental Data

Epileptic EEG data are used for the performance analysis by a calculating of the AUC from the segmented EEG signals, as AUC can distinguish normal, interictal and ictal forms. The AUC values range from 0.5 to 1 and reflect failed, poor, fair, good and excellent classification (Tape, 2019).

**Figure 5:**
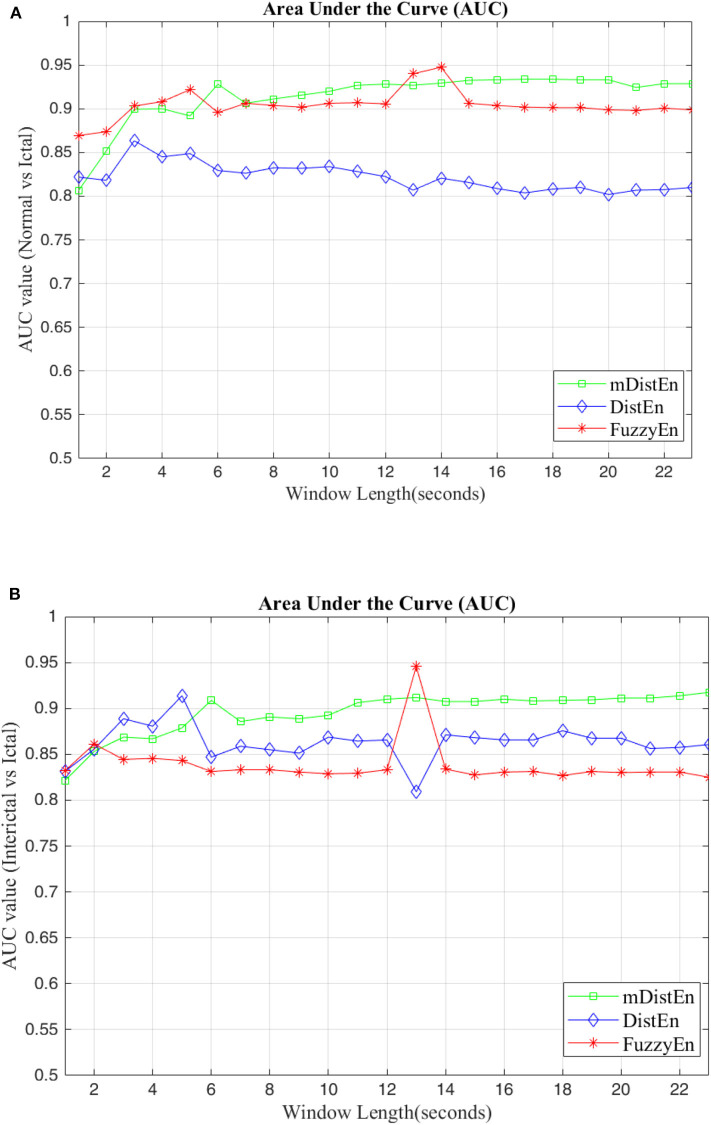
AUC values for different window lengths. **(A)** AUC values of the entropies for discrimination between normal and ictal and **(B)** between interictal and ictal.

**Figure 6:**
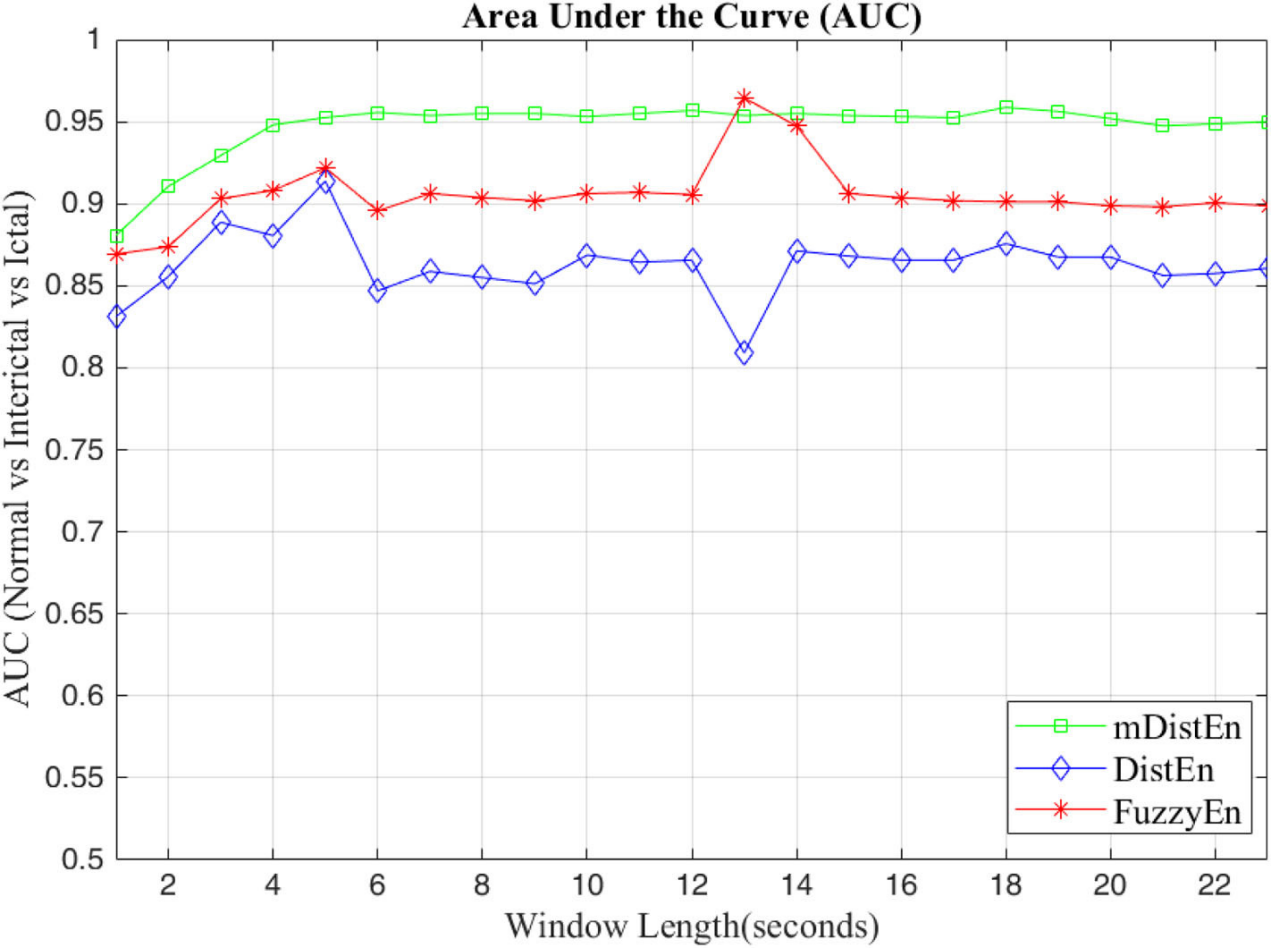
AUC values for the different window lengths for classifying normal from interictal and ictal signals.

First, EEG signals are divided into three groups: normal, interictal and ictal. The normal group includes datasets (A and B) and the interictal group includes datasets (C and D) and the ictal group contains dataset (E). Next, the AUC values are evaluated for 1-s segments of all 100 datasets from 2 to 23 s-segments along the data sample. Then, 5-fold cross-validation instead of 10-fold cross-validation (Acharya et al., 2015) is used to test the five datasets. When one dataset (A) is used as the testing data, the remaining four datasets (B-E) are used for training. This process is iterated until all five datasets (A–E) are used as the testing dataset. After, the entropy for all datasets are calculated, the AUC values are estimated based on the training dataset, and these values are plotted and shown in Figures 5, 6. Regarding Figure 5, the AUC values of mDistEn using Equation (8) are noticeably greater than those of the other two methods of fuzzy entropy and distribution entropy. Therefore, the mDistEn has better discriminatory power than the prior distribution entropies according to the AUC values, proving that mDistEn is sufficient considering both AUC and accuracy. Moreover, mDistEn is highly consistent compared with the previous distribution entropy. Performance is evaluated by calculating the sensitivity, specificity, and accuracy (Li et al., 2018):

(13) $$\begin{array}{l} \text{Specificity}=\frac{\text{TN}}{\text{TN}+\text{FP}}, \end{array}$$

(14) $$\begin{array}{l} \text{Sensitivity}=\frac{\text{TP}}{\text{TP}+\text{FN}}, \end{array}$$

(15) $$\begin{array}{l} \text{Accuracy}=\frac{\text{TP}+\text{TN}}{\text{TP}+\text{FP}+\text{TN}+\text{FN}}, \end{array}$$

where TP is the number of true positives and TN is the number of true negatives. These two values indicate correct labeling of the actual number of ictal and normal EEG signals by classifier. FP and FN are the number of false positives and false negatives which correspond to the number of ictal and normal signals that are incorrectly categorized by the classifier. It was already shown that the accuracy of the modified distribution entropy is slightly lower (by 1%) than that of FuzzyEn but greater than that of the previous distribution entropy as shown in Table 1. FuzzyEn yields an accuracy of 92% in the fifth run time with 13-s segments, while mDistEn with equation (8) and the distribution entropy get the accuracies of 91 and 86%, respectively, in the fifth run with 7-s segments and in the fourth run with 5-s segments.

**Table 1:** Performance evaluation of the different entropies.

**Entropy**	**Sensitivity (%)**	**Specificity (%)**	**Accuracy (%)**	**AUC (%)**
FuzzyEn	92.5	90	92	96
mDistEn	92.5	85	91	96
DistEn	83.75	95	86	91.3

Therefore, our new entropy is able to provide the promising accuracy with a small amount of input data, as well as optimal duration time (s) in the dataset. Consequently, short-duration input would lead to a good setting for the detect of epileptic seizures. However, entropy methods are still highly dependent on the prefix parameters and therefore one of the disadvantages of our proposed entropy. Further investigation on this issue would be suggested as our future work.

## Conclusion

In this paper, mDistEn is proposed for calculating the complexity of the time series data and was tested using both theoretical data and real-world EEG data. We proved that mDistEn is advantageous over fuzzy entropy and distribution entropy for distinguishing normal EEG data segments from epileptic EEG data segments, and for distinguishing the early state of seizures data (interictal period) from epileptic EEG data (ictal period). Moreover, our proposed entropy method can also discriminate normal EEG data from interictal EEG data and preictal state of EEG data from the ictal state of the EEG data. The results mentioned above are described in the calculation of AUC, which is most widely used for decision tasks. The mDistEn remains stable even when two new parameters are added. Furthermore, mDistEn yielded better accuracy than previous distribution entropy and only slightly lower accuracy than fuzzy entropy. Regarding the AUC values, mDistEn is able to distinguish early state of epilepsy from seizure onset, and thus, these parameters could be used to predict epileptic seizures. However, further studies are still needed to investigate the early detection of epilepsy.

## Data Availability Statement

Publicly available datasets were analyzed in this study. This data can be found here: http://epileptologie-bonn.de/cms/front_content.php?idcat=193&lang=3&changelang=3.

## Author Contributions

YW designed the contribution and supported for the technical knowledge. SA worked on conducting the experimental design, results, discussion, and the literature review.

## Conflict of Interest

The authors declare that the research was conducted in the absence of any commercial or financial relationships that could be construed as a potential conflict of interest.
